# Correction: IRE1–XBP1 pathway regulates oxidative proinsulin folding in pancreatic β cells

**DOI:** 10.1083/jcb.20170714303252019c

**Published:** 2019-03-29

**Authors:** Yuichi Tsuchiya, Michiko Saito, Hiroshi Kadokura, Jun-ichi Miyazaki, Fumi Tashiro, Yusuke Imagawa, Takao Iwawaki, Kenji Kohno

Vol. 217, No. 4, April 2, 2018. 10.1083/jcb.201707143.

In the third (HSP90(Cyt)) and fourth (HSP60(Mit)) panels of Fig. 1 B, the rightmost lane (pancreas tissue) was inadvertently trimmed during final preparation of the paper. The corrected version of Fig. 1 is shown below. This correction does not affect the interpretation of Fig. 1 B nor any of the conclusions in the paper.

**Figure 1.**
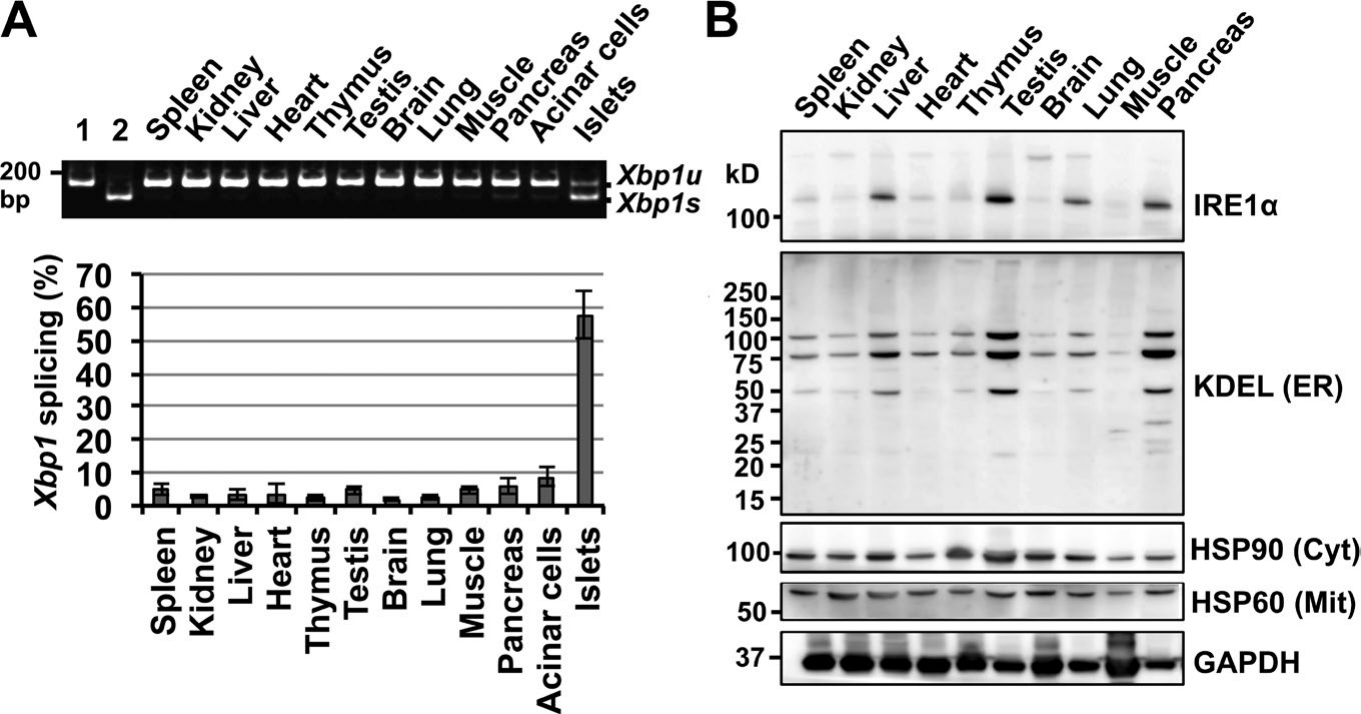
**Physiological activation of the IRE1α–XBP1 pathway in pancreatic islets. (A)** *Xbp1* mRNA splicing was analyzed by RT-PCR using total RNA isolated from the tissues of 8-wk-old WT male mice. The ratio of *Xbp1* mRNA splicing was quantified. Error bars show the means and SD. *n* = 3. *Xbp1* splicing (%) = *Xbp1s*/total *Xbp1* × 100. Lane 1, *Xbp1u*_pcDNA3.1(+). Lane 2, *Xbp1s*_pcDNA3.1(+). **(B)** Expression levels of IRE1α and ER-resident proteins harboring the KDEL motif (KDEL (ER)) in mouse tissues from an 8-wk-old WT male mouse based on immunoblotting. The expression levels of the cytosolic chaperone HSP90 (HSP90 (Cyt)) and the mitochondrial chaperone HSP60 (HSP60 (Mit)) were also examined. Positions of molecular mass markers are indicated on the left.

Both the HTML and PDF versions of the article have been corrected. This error appears only in print and in PDF versions downloaded on or before March 28, 2019.

